# The Human *Myotrophin* Variant Attenuates MicroRNA-Let-7 Binding Ability but Not Risk of Left Ventricular Hypertrophy in Human Essential Hypertension

**DOI:** 10.1371/journal.pone.0135526

**Published:** 2015-08-14

**Authors:** Yuyao Wang, Jingzhou Chen, Weihua Song, Yuxuan Wang, Yu Chen, Yu Nie, Rutai Hui

**Affiliations:** 1 Department of Biochemistry and Molecular Biology, Shanxi Medical University, Taiyuan, China; 2 Sino-German Laboratory for Molecular Medicine, State Key Laboratory of Cardiovascular Disease, Fuwai Hospital, National Center for Cardiovascular Diseases, Chinese Academy of Medical Sciences and Peking Union Medical College, Beijing, China; 3 Department of Thoracic Surgery, Shanxi Dayi Hospital, Taiyuan, China; University of Miami School of Medicine, UNITED STATES

## Abstract

Myotrophin, known as a myocardial hypertrophy-inducing factor, is responsible for the initiation of cardiac hypertrophy that transits to heart failure. MicroRNAs are small noncoding RNAs that down-regulate posttranscriptional expression of target molecules. We investigated the role of variants of the microRNA-binding site in *myotrophin* in affecting its expression and any association with cardiac hypertrophy. Bioinformatics demonstrated that variant rs17168525 was identified to be located in the let-7/miR-98-binding site of *myotrophin*. We further experimentally test to effects of the identified variant on *myotrophin* translation using luciferase reporter assay and Western blotting. We found that the C allele of rs17168525 suppressed myotrophin translation by facilitating let-7c binding, but not the T allele. Let-7c overexpression caused a significant decrease in the level of myotrophin protein. Next, we investigated the association of the variant with cardiac hypertrophy in 1614 hypertensive patients, including 552 with left ventricular hypertrophy and 1062 without left ventricular hypertrophy, as well as 591 healthy control subjects from a Han Chinese population. No significant association between the variant rs17168525 and left ventricular hypertrophy in hypertensive patients in a Han Chinese population (P>0.05). In conclusion, our experimental results provide evidence that the T allele of rs17168525 in the 3′-UTR of *myotrophin* might influence the level of myotrophin protein by interfering with let-7/miR-98 binding.

## Introduction

Cardiac hypertrophy is recognized as an adaptive process to a variety of physiological and pathological conditions [1]. Prolonged cardiac hypertrophy, a compensatory response that heart makes to maintain cardiac function, can induce a phase of phenotypic changes in which individual cardiomyocytes grow [2]. The changes in cellular phenotype are preceded and accompanied by a return to the fetal gene program [3]. However, cardiac hypertrophy may become aggravated by prolonged biomechanical stress and results in heart failure as well as sudden death [4,5]. Although previous studies have suggested that myocardium can release stress factors which contribute to the development of hypertrophy and heart failure [6], the underlying molecular mechanisms are still not clearly understood.

Myotrophin, a 12-kDa protein, is identified in spontaneously hypertensive rat hearts and cardiomyopathic human hearts [7]. *In vivo* and *in vitro* studies have shown that myotrophin plays a vital role in the initiation of cardiac hypertrophy that transits to heart failure [8–10]. The most recent evidence from clinical trials have observed an elevated concentration of plasma myotrophin among patients with heart failure [11] and those after acute myocardial infarction [12]. These data support that myotrophin may serve as an initiator of cardiac hypertrophy to stimulate the growth of cardiac myocytes.

Emerging evidence has indicated the importance of microRNAs (miRNAs) in the regulation of hypertrophic process [13–16]. MiRNAs are a class of small conserved noncoding RNA that negatively regulate target gene expression through complementarily binding to the 3′ untranslated region (3′-UTR) of mRNAs [17]. It inhibits the expression of specific genes by either degrading the target mRNA or direct translational inhibition [18]. The efficiency of binding of miRNAs to target transcripts depends on the sequence and intramolecular structure of the transcript. Single Nucleotide Polymorphisms (SNPs) occurring in the miRNA target sites can contribute to alterations in the structure of regions flanking them and thus influence the accessibility for miRNAs binding [19]. For example, Chen et al recently demonstrated that TT genotype (rs2507800) in the 3′-UTR of *angiopoietin-1* might reduce the risk of stroke by interfering with miR-211 binding efficiency [20].

Through a bioinformatics approach, we identified a variant, rs17168525, located in the let-7/miR-98 target site in the 3′-UTR of *myotrophin*. In the present study, we aimed to investigate whether the rs17168525 can modify the efficiency of let-7/miR-98 binding to *myotrophin* and then further contribute to the genetic susceptibility to cardiac hypertrophy.

## Materials and Methods

### Human *myotrophin* 3′-UTR luciferase constructs

To construct *myotrophin* 3′-UTR-luciferase reporter plasmids, a 46 bp sequence (Table A in S1 File) carrying either the wild type or the variant genotype of rs17168525 was synthesized and cloned into the pMIR-REPORT vector (Ambion, Austin, TX, USA), using restriction enzymes HindIII and SpeI. The reporter plasmid containing rs17168525C was defined as *myotrophin*-pMIR-C, and the one containing rs17168525T as *myotrophin*-pMIR-T. The resulting constructs (*myotrophin*-pMIR-C and *myotrophin*-pMIR-T) were verified by sequencing.

### Luciferase assay

Hela cells (1 × 10^5^ cells/well) were co-transfected with 2 μg of *myotrophin*-pMIR-C or *myotrophin*-pMIR-T plasmid, 0.01 μg of Renilla luciferase and 50 pmol of PremiR miRNA precursor of let-7c or PremiR Negative Control (Ambion), all combined with Lipofectamine 2000 (Invitrogen, Carlsbad, CA, USA). 48 h after transfection, the cells were washed and lysed with Passive Lysis Buffer (Promega, Madison, WI, USA), and the luciferase activities were measured using a luminometer (SIRIUS, Pforzheim, Germany).

### Primary culture of neonatal rat ventricular myocytes

The animal protocol was approved by the FuWai Hospital Animal Care and Use Committee and is in accordance with “Guide for the Care and Use of Laboratory Animals” published by the US National Institute of Health (National Institute of Health Publication No. 85–23, revised 1996). Neonatal rat ventricular myocytes were isolated and cultured from the ventricles of 1- to 2-day-old rats. In brief, left ventricular myocytes were dispersed from the ventricles by digestion with HEPES-buffered saline solution containing 0.06% collagenase. And then, cells were plated in DMEM with 10% fetal bovine serum, 0.1 mM bromodeoxyuridine (BrdU) at a density of 10^6^ cells/ml. Having been cultured for 48 h, the medium was replaced with a serum-free maintenance medium. Cells were incubated for another 24 h before any treatment.

### Western blot analysis

Cardiomyocytes were plated at a concentration of 10^6^ cells/ml. After transfection of PremiR miRNA precursor or Anti-miR miRNA inhibitor of let-7c for 48 h, the cells were lysed with Cell Lysis Buffer. We detected let-7c expression levels by using Taqman probe-based real-time PCR after treatment with PremiR miRNA precursor or Anti-miR miRNA inhibitor of let-7c. Equal amounts of proteins (5–30 μg) were subjected to SDS-PAGE and transferred to nitrocellulose membrane. The membrane was incubated with myotrophin antibody (Santa Cruz) or β-tublin (Cell Signaling) at 4°C overnight. Fluorescence-conjugated rabbit secondary monoclonal antibody (Cell Signaling) was added and incubated at room temperature for 1 h. Proteins were detected using the AP-NBT/BCIP system.

### Study population

Subjects were recruited from 24 communities in Xinyang, Henan Province, People’s Republic of China. The study was started in April 2005. All studies were approved by the Ethics Committee of Fuwai hospital. Signed informed consent was obtained from all participants, following a protocol that had been approved by the Ethics Committee of Fuwai hospital, and the study was conducted in accordance with the Declaration of Helsinki. The subjects consisted of 552 hypertensive patients (mean age 59.9±7.4 years) with LVH and 1062 patients (mean age 57.5±8.4 years) without LVH (Table 1). Hypertension was defined according to WHO (World Health Organization) criteria [21], as SBP (systolic BP) ≥140 mmHg and/or DBP (diastolic BP) ≥90 mmHg on average of two measurements or by current antihypertensive treatment. BPs were measured by the same investigator on the right arm using a mercury sphygmomanometer with standardized techniques after at least 5 min rest in a sitting position. Exclusion criteria included secondary arterial hypertension, atrioventricular conduction block, chronic obstructive bronchitis, bronchial asthma, chronic myeloproliferative diseases, diabetes, hypertrophic cardiomyopathy, valvular heart diseases, pulmonary hypertension, coronary heart disease and heart failure. Controls (n = 591, mean age 55.7±7.3 years) were recruited from age- and sex-matched healthy subjects in the same community, who had no history or symptoms of cardiovascular diseases.

**Table 1:** Baseline characteristics in controls and hypertensive patients.

Characteristics	Controls	Hypertensive patients	
		Without LVH	With LVH
**Subjects (n)**	591	1062	552
**Age (years)**	55.7±7.3	57.5±8.4*	59.9±7.4**
**Male (%)**	38.0	34.2	29.0
**BMI (kg/m** ^**2**^ **)**	23.5±3.2	25.1±3.5*	26.9±4.2*
**SBP (mmHg)**	118.1±10.8	158.2±21.2**	168.4±24.6**
**DBP (mmHg)**	78.5±6.7	97.2±10.3**	99.3±13.8**
**HDL-C (mmol/l)**	1.54±0.32	1.57±0.35	1.54±0.35
**LDL-C (mmol/l)**	2.78±0.82	3.07±0.85**	3.14±0.86**
**TC (mmol/l)**	5.18±1.08	5.46±1.09**	5.47±1.09**
**TG (mmol/l)**	1.44±1.06	1.66±1.56*	1.68±1.27**
**Glucose (mmol/l)**	5.27±1.70	5.58±1.77**	5.43±1.52*
**Cigarette smokers (%)**	24.1	18.0	16.1
**Alcohol consumers (%)**	22.0	22.7	18.1

Values are means ±SD or percentage.

*P <0.05 and

**P <0.01 compared with controls.

BMI, body mass index; BP, blood pressure; SBP, systolic BP; DBP, diastolic BP; HDL-C, high-density lipoprotein cholesterol; LDL-C, low-density lipoprotein cholesterol; TC, total cholesterol; TG, triacylglycerol.

### Clinical data collection and Echocardiography

Blood samples were taken after a 12 h overnight fasting. Plasma and urine biochemical variables were determined by using standard methods with an automatic analyzer (Hitachi 7060, Japan). All of the participants completed a questionnaire on current medication and life style. A complete medical history was obtained from all subjects, including family history of hypertension, diabetes mellitus and the following cardiovascular risk factors: alcohol intake, cigarette smoking, family history, weight, height, BMI (body mass index), SBP and DBP. All measurements and echocardiography were performed as described previously [22].

### Gene variant selection and genotyping

The variant rs17168525 was selected based on its relevance to gene expression, being located in the let-7/miR-98 target site of the 3′-UTR of *myotrophin*. Genomic DNA was extracted from peripheral blood using a method described previously [23]. The variant rs17168525 was genotyped by a ligase detection reaction (LDR) by the Shanghai Biowing Applied Biotechnology Co., Ltd. The primer and probe sequences and PCR and LDR product lengths of the variant are summarized in Table B in S1 File. Fragment amplification was carried out in 20 μl of multiplex PCR mixture containing 50 ng /μl of genomic DNA, 2 μl of 1× buffer, 0.6 μl of Mg^2+^, 2 μl of dNTPs, 0.3 μl of Taq polymerase, 4 μl of 1× Q-solution, 0.4 μl of primer mix and 9.7 μl of ddH_2_O. The PCR included initial denaturing at 95°C for 10 min, followed by 35 cycles of denaturing at 94°C for 30 s, annealing at 59°C for 90 s, and extension at 72°C for 1 min. The reaction was completed by a final extension at 72°C for 7 min. Reactions were performed on a thermal cycler Gene Amp PCR system 9600 (Perkin Elmer, Waltham, MA, USA). Further amplification was performed in a 10 μl volume of multiplex LDR reaction mixture, containing 1 μl (100 ng) of the resultant probe mix, 1 μl of probe mix, 0.05 μl of NEB Taq DNA ligase and 7.95 μl of ddH_2_O. The LDR conditions included initial denaturing at 95°C for 2 min, followed by 35 cycles of denaturing at 94°C for 30 s and annealing at 50°C for 2 min. LDR products (1 μl) were mixed with 1 μl of ROX (ABI, Foster City, CA, USA) and 1 μl of loading buffer, detected in an ABI PRISM 377 DNA Sequencer, and analyzed with Genemapper (ABI, Foster City, CA, USA). Reproducibility of genotyping was confirmed by sequencing in 400 randomly selected samples with 100% concordance.

### Statistical analysis

Values are expressed as means ± S.D. A χ^2^ test was used to test categorical variables, and the Hardy—Weinberg equilibrium was used to test the variant frequencies. One-way ANOVA was used for comparison of quantitative variables. Differences of quantitative variables between groups were analyzed using the Student′s t-test. ORs (odds ratios) and 95% CIs (confidence intervals) were calculated by use of multivariate logistic regression analyses, and were adjusted by age, gender, BMI, SBP, DBP, smoking status, alcohol consumption and glucose. Statistical analysis was performed with the SPSS 13.0 package. All significant tests were two-tailed and were considered significant at P<0.05.

## Results

### Variant rs17168525 C/T of *myothrophin* gene is in the let-7/miR-98 site

**Fig 1:**
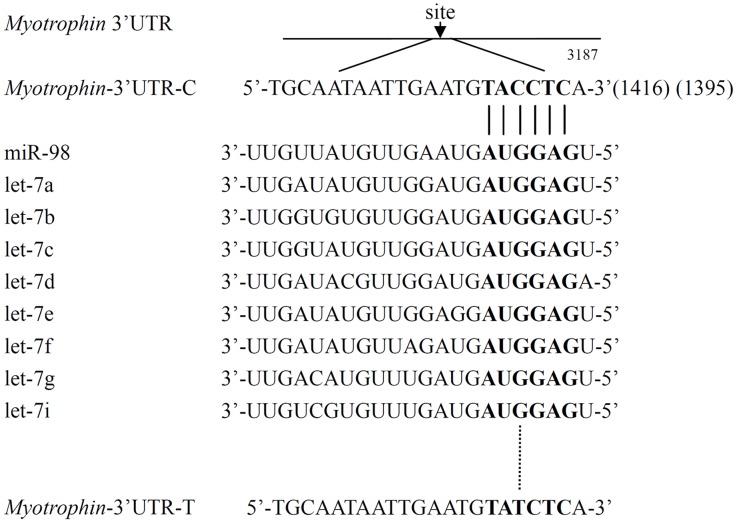
The *myotrophin* rs17168525 C/T variant occurs in the let-7/miR-98 binding site. The variant rs17168525 is a C to T change (mRNA sequence as reference) located in the predicted binding site for let-7/miR-98 in the 3′-UTR of the *myotrophin* gene. C-allele at rs17168525 base-paired with G in Watson—Crick mode (shown with a solid line). However, when the T-allele is present, base-pairing complementarity is interrupted (shown with a dashed line).

To search for miRNAs that might regulate human myotrophin expression, we used PicTar algorithm (http://pictar.bio.nyu.edu.) to screen potential miRNAs target sites in *myotrophin*, and discovered that variant rs17168525 occurs in the middle of the let-7/miR-98 complementarity seed binding sequence (Fig 1).

### The T-allele of *myotrophin* rs17168525 decreases ability of let-7c to regulate translation

**Fig 2:**
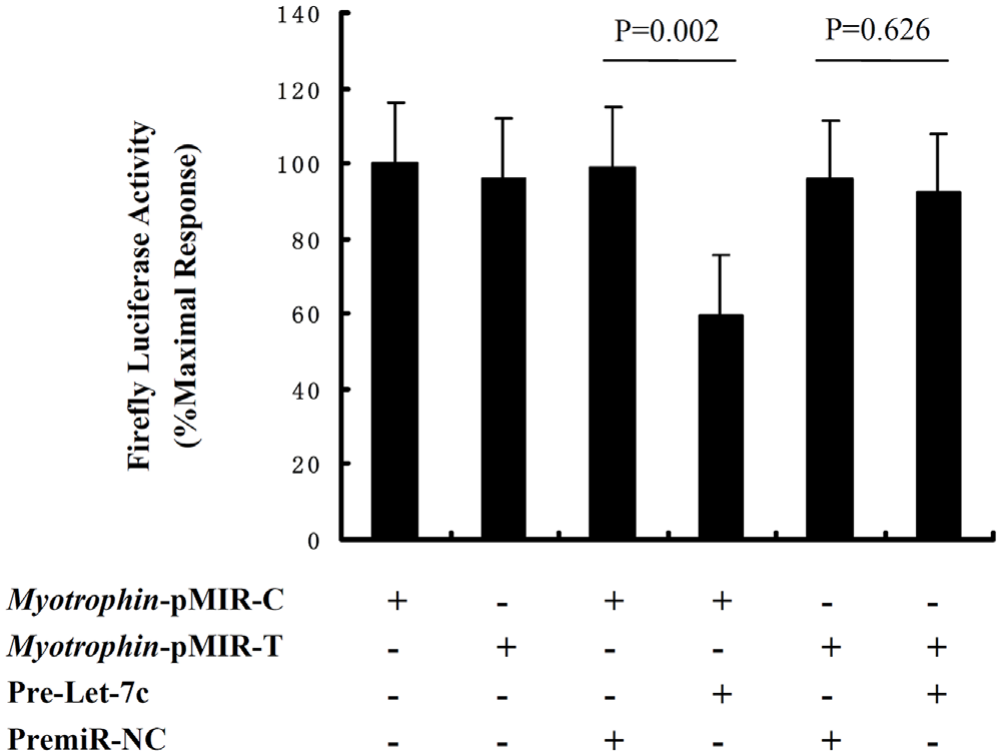
Testing the interaction between let-7c and *myotrophin* using a lucisferase reporter assay. Hela cells were co-transfected with *myotrophin*-pMIR-C or *myotrophin*-pMIR-T, and either negative control miRNA (PremiR-NC) or let-7c. 48 h after transfection, luciferase activities were measured. Firefly luciferase activity was normalized to *Renilla* luciferase expression, and mean activities ± S.E. from four independent experiments are shown. For *myotrophin*-pMIR-C transfection, PremiR-NC versus let-7c, P = 0.002; for *myotrophin*-pMIR-T transfection, PremiR-NC versus let-7c, P = 0.626.

We cloned a 46 bp sequence, comprising the predicted binding sequence around rs17168525, into the 3′-UTR of a luciferase reporter vector, pMIR-REPORTTM, carrying the wild-type (*myotrophin*-pMIR-C, with the C allele) and mutant sequence (*myotrophin*-pMIR-T, with the T allele) of rs17168525, respectively. It should be noted that miR-98 and some other members of the let-7 family have a very similar, if not identical, seed sequence and share target genes (Fig 1). Among the members of let-7 family, including miR-98, let-7c exhibited the highest minimal free energy of hybridization according to computational modeling [24]. We therefore investigated whether there was a relationship between the variant rs17168525 C/T and the let-7c binding site. Hela cells were co-transfected with these reporter constructs and with a PremiR miRNA, a precursor of let-7c (Pre-let-7c), or with a PremiR as a negative control (PremiR-NC). The *myotrophin*-pMIR-C reporter plus let-7c showed a significant reduction of *myotrophin*-pMIR-C signal (41% of the negative control; p<0.05) (Fig 2). This decrease was restored by co-transfection of let-7c with constructs *myotrophin*-pMIR-T reporter.

**Fig 3:**
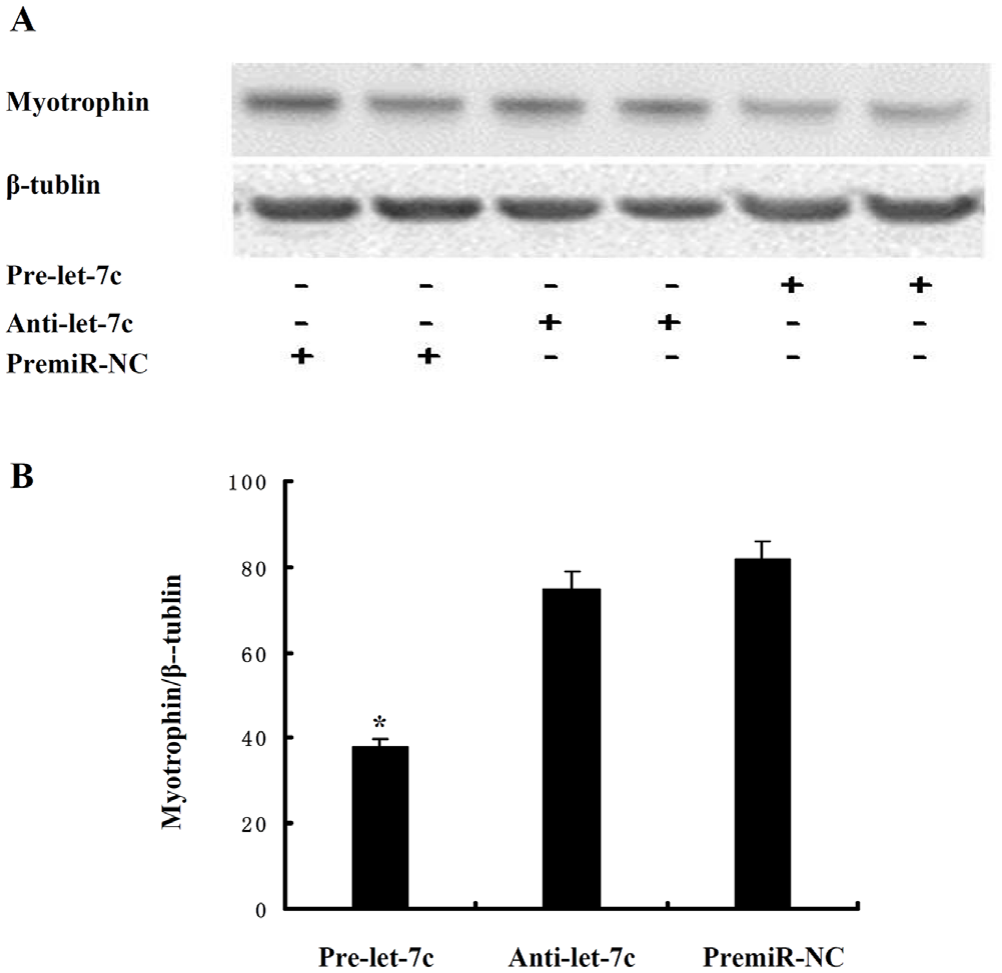
Let-7c suppresses the protein expression level of myotrophin *in vitro* cellular model. Cardiomyocytes were infected with PremiR miRNA precursor or Anti-miR miRNA inhibitor of let-7c (*A* and *B*). Myotrophin expression was analyzed by immunoblot 48 h after infection. *p < 0.05.

To examine whether let-7c down-regulates myotrophin in cardiomyocytes, we conducted western blotting to analyze myotrophin protein levels in primary neonatal rat cardiomyocytes with transfection of Pre-let-7c or Anti-miR miRNA inhibitor of let-7c (Anti-let-7c). Pre-let-7c increased the expression of let-7c (Fig A in S1 File). Likewise, we effectively down-regulated let-7c expression level by treating with Anti-let-7c (Fig A in S1 File). As indicated in Fig 3, let-7c overexpression caused a significant decrease in the level of the myotrophin protein. These results were consistent with the *in vitro* luciferase activity study.

### The T allele of variant rs17168525 is not associated with LVH risk

To test the hypothesis that the variant rs17168525 would modify genetic susceptibility, we investigated the association of variant rs17168525 with cardiac hypertrophy in a case-control study. The clinical characteristics of subjects are shown in Table 1. SBP, DBP and BMI, levels of triacyglycerol, total plasma cholesterol, glucose and LDL-C, were significantly higher in hypertensive patients without or with LVH compared with controls.

The distributions of the CC, CT and TT genotypes of the variant rs17168525 are summarized in Table 2. The distributions were in agreement with the Hardy-Weinberg equilibrium in both patients and controls. Genotype (CC/CT/TT) analyses were conducted for the dominant model (CT+TT compared with CC), additive model1 (TT compared with CC), additive model 2 (CT compared with CC) and the recessive model (TT compared with CC+CT). The frequency of T allele was 17.4% in hypertensive patients without LVH, 18.7% in hypertensive patients with LVH and 17.9% in controls. No differences existed using a χ^2^ test in hypertensive patients with LVH compared with either hypertensive patients without LVH or controls in either genotype frequency distribution (all p>0.05). In addition, the association between the variant rs17168525 and LVH was not significant in either men or women (Table C in S1 File).

**Table 2:** Possible associations between *myotrophin* variant and hypertension or left ventricular hypertrophy in the case-control study.

Groups (n)	MAF, %	P-value for	Genotype, n (%)				Model	Crude OR		Adjusted OR	Adjusted
		HWE				P-value^a^		(95%CI)	P-value	(95%CI)	P-value
	T		CC	CT	TT						
**Controls (591)**	17.9	0.404	401 (67.9)	168 (28.4)	22 (3.7)						
**Hypertensive patients (1614)**											
** Without LVH (1062)**	17.4	0.290	730 (68.7)	295 (27.8)	37 (3.5)	0.919		1.00		1.00	
** With LVH (552)**	18.7	0.827	366 (66.3)	166 (30.1)	20 (3.6)	0.852	Additive	0.96 (0.73–1.27)	0.79	0.95 (0.71–1.27)	0.73
							Dominant	0.90 (0.72–1.11)	0.32	0.91 (0.72–1.14)	0.39
							Recessive	0.96 (0.55–1.67)	0.89	0.97 (0.55–1.73)	0.92

^a^:Compared to controls. Adjusted ORs (95% CIs) were stratified by age, gender, BMI, SBP, DBP, smoking status, alcohol consumption and glucose.

Moreover, we also evaluated the relationship between rs17168525 polymorphism and echocardiographic variables by the general linear univariate model (Table D in S1 File). The echocardiographic parameters were not statistically different among the patients carrying the different genotypes (all p>0.05). In brief, individuals carrying the TT genotype or the T allele did not bear a greater risk of LVH in patients with hypertension than those carrying the CC genotype and the C allele, based on logistic regression for adjustment to confounding parameters (Table 2).

## Discussion

In the present study, we found that the TT genotype of variant rs17168525 in *myotrophin* was resistant to let-7/miR-98-induced down-regulation of myotrophin, while there was no significant association between this polymorphism and left ventricular hypertrophy in a Chinese Han population.

To date, different computational approaches have been provided a mapping of all known SNPs onto a set of bioinformatic predicted miRNA target sites [25,26], including rs17168525 resides within the target site for let-7 family within the 3’-UTR of the gene *myotrophin* [27]. However, a small portion of these predicted target sites have been experimentally validated [28]. Notably, the critical region for a miRNA is nucleotides 2–7 from the 5’ end of the miRNA, called as “seed” region, which most often binds to a target site in the 3’-UTR of the given mRNA by perfect Watson-Crick complementarity [29]. Variants in miRNA target sites have the potential to alter the base-pairing patterns which in turn would affect the accessibility of the miRNA at the target site and gene activity [19]. In this study, computer alignment revealed that variant rs17168525 located in the let-7/miR-98 complementarity seed binding sequence. We processed luciferase reporter assay and found when the C-allele was present in luciferase mRNAs, the ability of let-7c to inhibit luciferase activity was significantly attenuated that were comparable with experiments utilizing the constructs harboring T-allele and let-7c. One limitation is that we did not set a known let-7 target as a positive control in western blot analysis. However, our results were verified by western blotting test to analyze myotrophin protein levels in primary neonatal rat cardiomyocytes. Let-7c overexpression led to a significant decrease in the level of the myotrophin protein compared to utilize the Anti-let-7c. Taken together, these experiments indeed demonstrated that when seed sequence complementarity was not fulfilled, myotrophin levels were always higher than the levels obtained when perfect complementarity was present between the let-7/miR-98 and the target site in *myotrophin*.

On the basis of candidate gene strategy as well as previous functional findings, we further investigated the association of this variant with cardiac hypertrophy in a community-based cohorts study in Han Chinese population. No significant difference was observed in the genotype frequency distribution among three groups. Previous studies have reported a gender difference in myotrophin plasma levels in HF patients [11]. Thus, we explored the genotype frequency distribution in different subgroups according to gender, but no positive results were found. In addition, the echocardiographic parameters were not statistically different among different genotypes. These results suggest that the variant rs17168525 of *myotrophin* gene is not significantly associated with LVH in Han Chinese population. The disparity between our genetic association study findings and function study findings can be partially explained by the following reasons.

First, genetic heterogeneity of population might explain the discrepant results. Different geographical and racial backgrounds of the individuals can affect the consequences of an association study [30]. Ethnicity and population composition may strongly influence the prevalence of *myotrophin* gene polymorphisms. On the basis of the HapMap database, the minor allele frequency (MAF) of rs17168525 was 0.008 for CEPH, 0.089 for JPT and 0.189 for Han Chinese in Beijing. Consistently, our present study showed that the MAF of rs17168525T allele was 0.179 for Han Chinese in Xinyang region, but higher than that registered for CEPH and JPT. These data imply that the variant rs17168525 might have different effects on cardiac hypertrophy among different populations. Such a discrepancy may be caused by distinct ethnicity-related factors that affect the levels of myotrophin production.

Secondly, cardiac hypertrophy is known as a complex process affected by both genetic and environmental risk factors, and other variants in or near the *myotrophin* gene may exert important genetic effects on left ventricular hypertrophy risk. To the best of our knowledge, the present study is the first investigation to test the relationship between the *myotrophin* polymorphism and cardiac hypertrophy. Of note, gene-gene and gene-environment interactions have a larger impact on genetic susceptibility than the independent effects of each locus [31,32], which may account for the absence of associations observed in our population.

Thirdly, polymorphisms, either at the target site or around the target site of miRNA binding, might influence the secondary structure at the target site. A most recent study showed that a C-to-T polymorphism, near the miR-24 binding site on DHFR gene, can result in DHFR overexpression [33]. In this case, other variants in *myotrophin* gene may have potential functions to alter the secondary structure which in turn would determine the accessibility of the let-7/miR-98 binding at the target site.

Finally, another possible reason for the disparity is that human may have multiple transcripts of myotrophin. Previous report demonstrated six myotrophin transcripts in SHR heart and the levels of all the transcripts are significantly elevated in SHR hearts compared with levels of the same transcript in WKY rat hearts [34]. Thus it is possible that human *myotrophin* variant rs17168525 may be only a part of the whole expression pool of myotrophin and the effect of human *myotrophin* variant rs17168525 could be diluted.

There are several limitations in the present study. First, the function of let-7 family is largely unknown in heart, although a very recent study demonstrated that let-7/miR-98 negatively regulates cardiac hypertrophy [35]. Similarly, our *in vitro* study suggests that let-7/miR-98 can inhibit the expression of myotrophin, but its role *in vivo* remains to be further investigated. Also, the mechanisms by which the binding of let-7/miR-98 regulates cardiac hypertrophy *in vivo* still need to be investigated. In addition, we carried out a community-based association study between variant rs17168525 and left ventricular hypertrophy only in Han Chinese population. Further studies are needed to elucidate the role of this polymorphism in the pathogenesis of cardiac hypertrophy in various ethnic groups.

Another limitation of the present study is that we did not have the data about the expression level of myotrophin in serum/plasma from control and hypertensive patients, thus, the association between myotrophin and development of left ventricular hypertrophy in human essential hypertension can not be well established. However, several previous studies showed that elevation of myotrophin in the plasma of patients with HF, particularly in males [11] and the myotrophin levels in patients with AMI were significantly higher than those observed in the control subjects [12]. Further measurement of myotrophin expression level is needed in the future studies.

In summary, we report here that T allele of rs17168525 in the 3′-UTR of *myotrophin* might increase the risk of cardiac hypertrophy by interfering with let-7/miR-98 binding. The lack of association was found between the polymorphism and left ventricular hypertrophy in a Han Chinese population. It will be important to detect mechanisms underlying the effect of this variant or other variants in or near miRNA binding target site of *myotrophin* and true association between *myotrophin* polymorphisms with cardiac hypertrophy.

## Supporting Information

S1 File Supplementary tables and figures. A DOC file containing further tables and figures described in this article. (DOC)
(DOC)
