# Correction: The Human *Myotrophin* Variant Attenuates MicroRNA-Let-7 Binding Ability but Not Risk of Left Ventricular Hypertrophy in Human Essential Hypertension

**DOI:** 10.1371/journal.pone.0146735

**Published:** 2016-01-05

**Authors:** PLOS ONE Staff

Dr. Zhidong Ye is not included in the author byline. He should be listed as the seventh author and affiliated with the Department of Cardiovascular Surgery, China-Japan Friendship Hospital, Beijing, China. The contributions of this author are as follows: Performed the experiments, analyzed the data, and contributed reagents/materials/analysis tools.

The incorrect version of Fig 3 appears in the paper. Please see the correct version of Fig 3 here. The publisher apologizes for the error.

**Fig 3:**
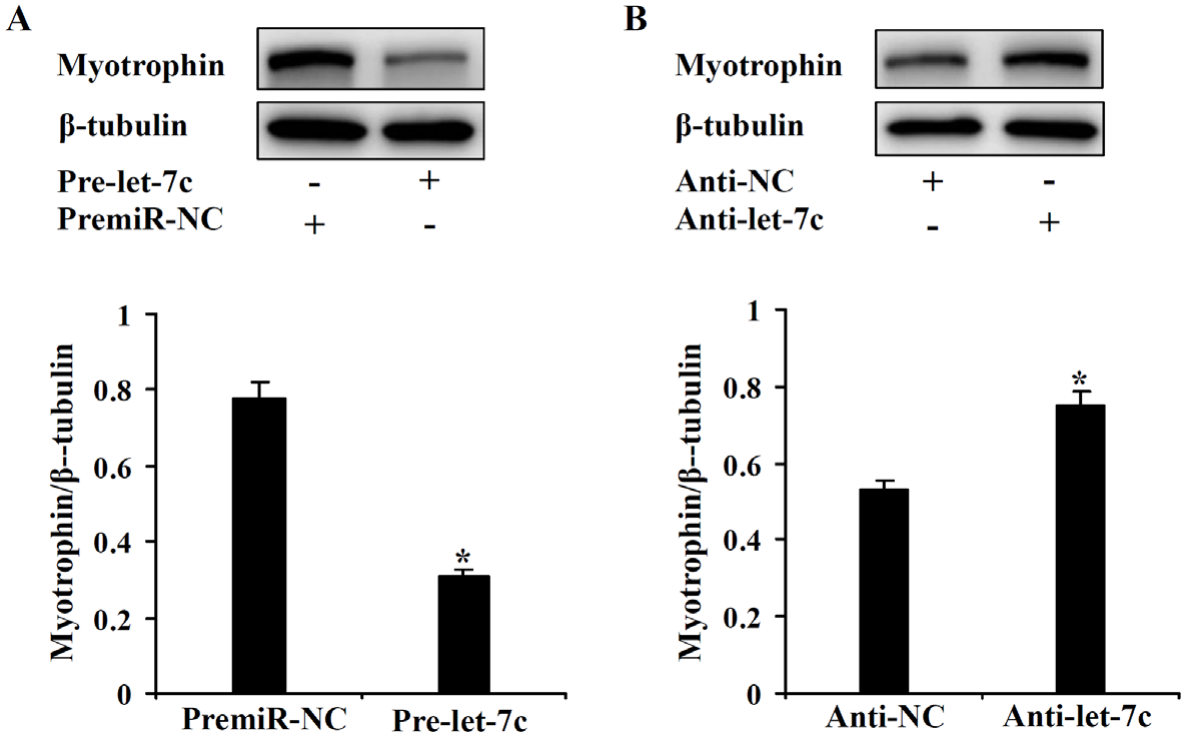
Let-7c suppresses the protein expression level of myotrophin *in vitro* cellular model. Cardiomyocytes were infected with PremiR miRNA precursor or Anti-miR miRNA inhibitor of let-7c (*A* and *B*). Myotrophin expression was analyzed by immunoblot 48 h after infection. *p < 0.05.

## References

[1] Wang Y, Chen J, Song W, Wang Y, Chen Y, Nie Y, et al (2015) The Human *Myotrophin* Variant Attenuates MicroRNA-Let-7 Binding Ability but Not Risk of Left Ventricular Hypertrophy in Human Essential Hypertension. PLoS ONE 10(8): e0135526 doi: 10.1371/journal.pone.0135526 26274321 10.1371/journal.pone.0135526 PMC4537090

