# Supplementary material for: The Human Myotrophin Variant Attenuates MicroRNA-Let-7 Binding Ability but Not Risk of Left Ventricular Hypertrophy in Human Essential Hypertension
Source: PLoS One. 2015 Aug 14;10(8):e0135526. doi: 10.1371/journal.pone.0135526 (PMC4537090; doi:10.1371/journal.pone.0135526)
Supplement: S1 File — A DOC file containing further tables and figures described in this article. (DOC) (DOC) [file pone.0135526.s001.doc]

**Supplementary tables and figures**

The human *myotrophin* variant attenuates microRNA-let-7 binding ability but not risk of left ventricular hypertrophy in human essential hypertension

Yuyao Wang, Jingzhou Chen, Weihua Song, Yuxuan Wang, Yu Chen, Yu Nie, Rutai Hui

Correspondence:

Yuyao Wang, PhD, Department of Biochemistry and Molecular Biology, Shanxi Medical University, 56 South Road of Xinjian, Taiyuan 030001, PR China. Tel.: +86 351 4135670; Fax: +86 351 4135670. E-mail addresses: [wyybio@163.com](mailto:wyybio@163.com).

Rutai Hui, MD, PhD, FuWai Hospital, Chinese Academy of Medical Sciences and Peking Union Medical College, 167 Beilishi Road, Beijing 100037, PR China. Tel.: +86 10 88398154; Fax: +86 10 68331730. E-mail addresses: [huirutai@gmail.com](mailto:huirutai@gmail.com).

**Table A.** **The sequences of insertion into pMIR-REPORT vector contained variant rs17168525.**

| Variant | Sequences |
| --- | --- |
| rs17168525C | 5’-TATTAAGACCAAGTCATGCAATAATTGAATGTA**C**CTCAAATTTTTA-3’ |
| rs17168525T | 5’-TATTAAGACCAAGTCATGCAATAATTGAATGTA**T**CTCAAATTTTTA-3’ |

**Table B. Primer and probe sequence and PCR and LDR product length of variant rs17168525.**

| Variable | Sequence (5’-3’) | PCR or LDR length (bp) |
| --- | --- | --- |
| Primer |  | 236 |
| rs17168525-up | TTGCCTTTAAAAACCAATTCAA |  |
| rs17168525-low | ATTTCCAAATATTTATTTCATGAGC |  |
| Probe |  |  |
| rs17168525_modify | P-CTCAAATTTTTAGGGGAGGGTGGGTTTTTTTTTTTTTTTTT-FAM |  |
| rs17168525_A | TTTTTTTTTTTTTTTTCAAGTCATGCAATAATTGAATGTAT | 82 |
| rs17168525_G | TTTTTTTTTTTTTTTTTTCAAGTCATGCAATAATTGAATGTAC | 84 |

PCR, polymerase chain reaction; LDR, ligase detection reaction.

**miR-let-7c/U6**

**miR-let-7c/U6**

**(A)**

**(B)**

*****

*****

**Fig A. Taqman probe-based real-time PCR was used to ananlyze let-7c expression levels after treatment with Pre-let-7c or Anti-let-7c (*A* and *B*).** *p < 0.05.

**Table C. Distribution of the variant rs17168525 in the controls, hypertensive patients and hypertensive subgroups by gender.**

| Groups (n) | Genotypes frequencies (%) | | | p-calue^a^ |
| --- | --- | --- | --- | --- |
|  | CC | CT | TT |  |
| Controls (591) |  |  |  |  |
| Male (225) | 146 (64.9) | 70 (31.0) | 9 (4.1) |  |
| Female (366) | 254 (69.4) | 99 (27.0) | 13 (3.6) |  |
| Hypertensive patients (1614) |  |  |  |  |
| Without LVH (1062) |  |  |  |  |
| Male (363) | 247 (68.1) | 101 (27.8) | 15 (4.1) | 0.750 |
| Female (699) | 483 (69.1) | 194 (27.8) | 22 (3.1) | 0.915 |
| With LVH (552) |  |  |  |  |
| Male (160) | 105 (65.6) | 48 (30.0) | 7 (4.4) | 0.976 |
| Female (392) | 261 (66.6) | 118 (30.1) | 13 (3.3) | 0.660 |

LVH, left ventricular hypertrophy. ^a^Compared to controls by gender.

**Table D. Echocardiographic variables in controls and hypertensive patients according to the *myotrophin* rs17168525 genotype.**

| Variable | Controls (n=591) | | | Hypertensive patients without LVH (n=1062) | | | Hypertensive patients with LVH (n=552) | | |
| --- | --- | --- | --- | --- | --- | --- | --- | --- | --- |
|  | CC | CT | TT | CC | CT | TT | CC | CT | TT |
| n | 401 | 168 | 22 | 730 | 295 | 37 | 366 | 166 | 20 |
| IVSd | 8.6±1.1 | 8.6±1.1 | 8.5±1.3 | 9.2±1.3 | 9.2±1.3 | 9.4±1.1 | 10.8±1.4 | 11.1±1.5 | 10.9±1.5 |
| LVEDD | 43.8±4.3 | 44.2±4.3 | 43.1±4.2 | 43.7±4.4 | 43.6±4.1 | 43.0±4.4 | 48.6±4.6 | 48.4±4.6 | 48.0±5.2 |
| LVESD | 27.6±4.4 | 28.2±4.4 | 26.2±3.3 | 28.1±4.5 | 28.1±4.3 | 27.6±4.7 | 31.7±6.0 | 31.6±5.7 | 30.6±8.3 |
| LVPW | 8.7±1.2 | 8.6±1.0 | 8.5±1.1 | 9.1±1.1 | 9.0±1.1 | 9.1±1.0 | 11.0±3.0 | 10.9±2.1 | 11.5±4.1 |
| RWT (%) | 39.8±6.0 | 39.2±6.0 | 39.5±5.2 | 42.3±7.3 | 42.2±7.1 | 43.6±7.3 | 45.2±9.0 | 45.9±8.7 | 47.2±11.3 |
| LVMI (g/m^2^) | 35.0±6.6 | 34.4±6.4 | 33.7±6.7 | 37.1±6.1 | 37.0±6.4 | 36.6±6.8 | 58.5±13.0 | 58.3±10.0 | 59.0±8.5 |

Results are mean ± SD. A general linear univariate model was performed, considering each cardiac echocardiographic variable as a dependent variable, with covariates including age, gender, BMI, SBP, DBP, glucose, HDL-C, LDL-C, triacylglycerol and total plasma cholesterol. A Bonferroni test was performed for adjustment of multiple comparisons. LV, left ventricular; IVSd, interventricular septal thickness; LVEDD, LV end-diastolic diameter; LVESD, LV end-systolic diameter; LVPW, LV posterior wall; RWT, relative wall thickness; LVMI, LV mass index.
